# Clinical Global Assessment Scale (CGAS) Recording at Admission and Discharge for Young People Admitted to a General Adolescent Inpatient unit

**DOI:** 10.1192/bjo.2026.11674

**Published:** 2026-06-30

**Authors:** Chris Popoola, James McClung, Praveen Kumar, Laura Sutherland

**Affiliations:** 1 University of Dundee, Dundee, United Kingdom; 2 Imperial College London, London, United Kingdom; 3 NHS Grampian, Aberdeen, United Kingdom; 4 NHS Tayside, Dundee, United Kingdom

## Abstract

**Aims:**

Tier 4 CAMHS general adolescent inpatient services are required to monitor clinical outcomes using measures including CGAS, collected on admission and at discharge. This audit aims to evaluate the completeness of CGAS recording at admission and discharge, and describe CGAS distribution for young people admitted to a general adolescent inpatient unit over a nine-month period.

**Methods:**

Data was collected over a nine-month period (30/01/2025 to 30/09/2025), giving n=50 admissions for analysis. Inclusion criteria was all completed admissions within the audit period, and exclusion criteria was any entries where the episode was incomplete or duplicated. For each admission, presence/absence of CGAS at admission, presence/absence of CGAS at discharge, CGAS score at admission (banded into 10-point ranges), and CGAS score at discharge (similarly banded) were extracted from patients’ admission forms and discharge summaries on MORSE healthcare electronic data.

**Results:**

CGAS was recorded on admission for 39/50 admissions. The admission CGAS completion rate of 78.0% did not meet the local target of ≥90%. CGAS was recorded on discharge for 29/50 admissions. The discharge CGAS completion rate of 58.0% was well below the local target of ≥90%. 56% had admission CGAS scores in the 31–40 band, indicating marked impairment in functioning. 16% scored 21–30, and 4% scored 11–20, reflecting very severe impairment. By discharge, a greater proportion of young people had scores in the 41–50 and 51–60 bands, and a small number reached 61–70 and 71–80, suggesting improvement in functioning for those with recorded scores. However, because discharge CGAS was missing or “TBD” for almost half of the sample, these figures may over-represent young people whose episodes were more complete or stable at discharge.

**Conclusion:**

Where recorded, CGAS distributions indicate clinically meaningful improvement in functioning from admission to discharge, but missing discharge data in 21/50 cases weakens the ability to demonstrate outcomes for the whole cohort. Going forward, the Information Officer will routinely check discharge summaries (where no CGAS score is included, the author will be contacted) and the Medical Secretary will only accept discharge summaries that include a completed CGAS, querying any summaries where this is missing. A re-audit will be undertaken for all eligible admissions from 10/2025 to 02/26, using the same criteria and targets to assess whether CGAS completion at admission and discharge has improved towards or beyond the ≥90% threshold.

